# Supplementary material for: Spatial Risk Factors of Vector-Borne Diseases in Pacific Island Countries and Territories: A Scoping Review
Source: Trop Med Infect Dis. 2025 Dec 24;11(1):6. doi: 10.3390/tropicalmed11010006 (PMC12846491; doi:10.3390/tropicalmed11010006)
Supplement: Supplementary file 1 [file tropicalmed-11-00006-s001.zip › Supplementary File S2_Search strategy.pdf]

SEARCH STRATEGY

PUBMED
("Vector Borne Diseases"[Mesh] OR “Vector-borne disease”[Title/Abstract] OR "Arbovirus Infections"[Mesh] OR "Mosquito-Borne Diseases"[Mesh] OR "Tick-Borne Diseases"[Mesh] OR “Outbreak”[Title/Abstract] OR "Malaria"[Title/Abstract] OR “Dengue”[Title/Abstract] OR “Zika”[Title/Abstract] OR "Chikungunya”[Title/Abstract] OR "Yellow fever"[Title/Abstract] OR “Lymphatic Filariasis"[Title/Abstract] OR “Chagas Disease"[Title/Abstract] OR “Trypanosomiasis"[Title/Abstract] OR “Schistosomiasis"[Title/Abstract] OR “Onchocerciasis"[Title/Abstract] OR “Tungiasis"[Title/Abstract] OR “Ross river”[Title/Abstract] OR “Japanese encephalitis”[Title/Abstract] OR “Leishmaniasis”[Title/Abstract])
AND<br>(“Climate change*"[Title/Abstract] OR "Climate"[Mesh] OR “Climate"[Title/Abstract] OR "Environment"[Mesh] OR “Environment*"[Title/Abstract] OR “climate conditions"[Title/Abstract] OR "Weather"[Mesh] OR “weather"[Title/Abstract] OR “Global warming”[Title/Abstract] OR “Globalization”[Title/Abstract] OR “Temperature"[Title/Abstract] OR “humidity” OR “Precipitation"[Title/Abstract] OR “Rainfall”[Title/Abstract] OR “Flooding"[Title/Abstract] OR “Deforestation”[Title/Abstract] OR “Aforestation”[Title/Abstract] OR “Land cover”[Title/Abstract] OR “Vegetation”[Title/Abstract] OR “el Nino”[Title/Abstract] OR “la Nina”[Title/Abstract] OR “Extreme climate events”[Title/Abstract] OR “Urbanization”[Title/Abstract] OR “Crowding”[Title/Abstract] OR “Migration"[Title/Abstract] OR “Income"[Title/Abstract] OR “Education”[Title/Abstract] OR “Unemployment”[Title/Abstract] OR “Access”[Title/Abstract] OR “Accessible”[Title/Abstract] OR “Accessibility”[Title/Abstract] OR “Availability”[Title/Abstract] OR "Healthcare provision"[Title/Abstract] OR "Healthcare infrastructure"[Title/Abstract] OR "Healthcare service delivery"[Title/Abstract] OR "Healthcare utilization"[Title/Abstract] OR "Healthcare utilisation"[Title/Abstract] OR "Health service utilization"[Title/Abstract] OR "Health service utilisation"[Title/Abstract] OR “Driver*”[Title/Abstract] OR “indicator*”[Title/Abstract] OR “factor*”[Title/Abstract] OR “characteristic*”[Title/Abstract] OR “determinant*”[Title/Abstract] OR “predictor*”[Title/Abstract])
AND<br>("Pacific Islands"[Mesh] OR “Pacific Islands”[Title/Abstract] OR “Melanesia”[Title/Abstract] OR “Fiji”[Title/Abstract] OR “Papua New Guinea”[Title/Abstract] OR “Solomon Islands”[Title/Abstract] OR “Vanuatu”[Title/Abstract] OR “New Caledonia”[Title/Abstract] OR “Polynesia”[Title/Abstract] OR “Cook Islands”[Title/Abstract] OR “Independent state of Samoa”[Title/Abstract] OR “Tonga”[Title/Abstract] OR “Tuvalu”[Title/Abstract] OR “Niue”[Title/Abstract] OR “Tokelau”[Title/Abstract] OR “Kiribati”[Title/Abstract] OR “Micronesia”[Title/Abstract] OR “Federated States of Micronesia”[Title/Abstract] OR “Marshall Island”[Title/Abstract] OR “Palau”[Title/Abstract] OR “Narau”[Title/Abstract] OR “French Polynesia”[Title/Abstract] OR “Wallis and Futuna”[Title/Abstract] OR “American

Samoa"[Title/Abstract] OR "Guam"[Title/Abstract] OR "Northern Mariana Islands"[Title/Abstract] OR "Pitcairn Islands"[Title/Abstract] OR "Norfolk Island"[Title/Abstract] OR "Christmas Island"[Title/Abstract] OR "Cocos Islands"[Title/Abstract] OR "United States Minor Outlying Islands"[Title/Abstract])
**TOTAL RESULTS**
1137 Articles

**SCOPUS**
(TITLE ("vector-borne disease*") OR TITLE-ABS ("Arbovirus infections" OR " Mosquito-borne diseases" OR "Tick-borne diseases" OR "Outbreak" OR "Malaria" OR "Dengue" OR "Zika" OR "Chikungunya" OR "Yellow fever" OR "Lymphatic Filariasis" OR "Chagas Disease" OR "Trypanosomiasis" OR "Schistosomiasis" OR "Onchocerciasis" OR "Tungiasis" OR "Ross river" OR "Japanese encephalitis" OR "Leishmaniasis"))
AND (TITLE-ABS ("Climate" OR "Climate change*" OR "Environment*" OR "climate conditions" OR "weather" OR "Global warming" OR "Globalization" OR "Temperature" OR "humidity" OR "Precipitation" OR "Rainfall" OR "Flooding" OR "Deforestation" OR "Aforestation" OR "Land cover" OR "Vegetation" OR "el nino" OR "la Nina" OR "Extreme climate events" OR "Urbanization" OR "Crowding" OR "Migration" OR "Income" OR "Education" OR "Unemployment" OR "access" OR "accessible" OR "accessibility" OR "availability" OR "Healthcare provision" OR "Healthcare infrastructure" OR "Healthcare service delivery" OR "Healthcare utilization" OR "Healthcare utilisation" OR "Health service utilization" OR "Health service utilisation" OR "driver*" OR "indicator*" OR "factor*" OR "characteristic*" OR "determinant*" OR "predictor*"))
AND (TITLE-ABS ("Pacific Islands" OR "Melanesia" OR "Fiji" OR "Papua New Guinea" OR "Solomon Islands" OR "Vanuatu" OR "New Caledonia" OR "Polynesia" OR "Cook Islands" OR "Independent state of Samoa" OR "Tonga" OR "Tuvalu" OR "Niue" OR "Tokelau" OR "Kiribati" OR "Micronesia" OR "Federated States of Micronesia" OR "Marshall Island" OR "Palau" OR "Narau" OR "French Polynesia" OR "Wallis and Futuna" OR "American Samoa" OR "Guam" OR "Northern Mariana Islands" OR "Pitcairn Islands" OR "Norfolk Island" OR "Christmas Island" OR "Cocos Islands" OR "United States Minor Outlying Islands"))
**TOTAL RESULTS**
881 Articles

WEB OF SCIENCE
(TS=(("vector-borne disease" OR TITLE-ABS “Arbovirus infections” OR “ Mosquito-borne diseases” OR “Tick-borne diseases” OR “Outbreak” OR “Malaria” OR “Dengue” OR “Zika” OR “Chikungunya” OR “Yellow fever” OR “Lymphatic Filariasis” OR “Chagas Disease” OR “Trypanosomiasis” OR “Schistosomiasis” OR “Onchocerciasis” OR “Tungiasis” OR “Ross river” OR “Japanese encephalitis” OR “Leishmaniasis”)))
AND (TS=((“Climate” OR “Climate change*” OR “Environment*” OR “climate conditions” OR “weather” “Global warming” OR “Globalization” OR “Temperature” OR “humidity” OR “Precipitation” OR “Rainfall” OR “Flooding” OR “Deforestation” OR “Aforestation” OR “Land cover” OR “Vegetation” OR “el nino” OR “la Nina” OR “Extreme climate events” OR “Urbanization” OR “Crowding” OR “Migration” OR “Income” OR “Education” OR “Unemployment” OR “access” OR “accessible” OR “accessibility” OR “availability” OR "Healthcare provision" OR "Healthcare infrastructure" OR "Healthcare service delivery" OR "Healthcare utilization" OR "Healthcare utilisation" OR "Health service utilization" OR "Health service utilisation" OR “driver*” OR “indicator*” OR “factor*” OR “characteristic*” OR “determinant*” OR “predictor*”)))
AND (TS=((“Pacific Islands” OR “Melanesia” OR “Fiji” OR “Papua New Guinea” OR “Solomon Islands” OR “Vanuatu” OR “New Caledonia” OR “Polynesia” OR “Cook Islands” OR “Independent state of Samoa” OR “Tonga” OR “Tuvalu” OR “Niue” OR “Tokelau” OR “Kiribati” OR “Micronesia” OR “Federated States of Micronesia” OR “Marshall Island” OR “Palau” OR “Narau” OR “French Polynesia” OR “Wallis and Futuna” OR “American Samoa” OR “Guam” OR “Northern Mariana Islands” OR “Pitcairn Islands” OR “Norfolk Island” OR “Christmas Island” OR “Cocos Islands” OR “United States Minor Outlying Islands”)))
**TOTAL RESULTS**
990 Articles
